# Vestibular/ocular motor symptoms in concussed adolescents are linked to retrosplenial activation

**DOI:** 10.1093/braincomms/fcac123

**Published:** 2022-05-13

**Authors:** Anna Manelis, João Paulo Lima Santos, Stephen J. Suss, Cynthia L. Holland, Richelle S. Stiffler, Hannah B. Bitzer, Sarrah Mailliard, Madelyn A. Shaffer, Kaitlin Caviston, Michael W. Collins, Mary L. Phillips, Anthony P. Kontos, Amelia Versace

**Affiliations:** 1 Department of Psychiatry, University of Pittsburgh, Pittsburgh, PA, USA; 2 Department of Orthopaedic Surgery/UPMC Sports Medicine Concussion Program, University of Pittsburgh, Pittsburgh, PA, USA; 3 Department of Radiology, Magnetic Resonance Research Center, University of Pittsburgh Medical Center, University of Pittsburgh, Pittsburgh, PA, USA

**Keywords:** concussion, adolescents, vestibular/ocular motor symptoms, working memory, retrosplenial cortex

## Abstract

Following concussion, adolescents often experience vestibular and ocular motor symptoms as well as working memory deficits that may affect their cognitive, academic and social well-being. Complex visual environments including school activities, playing sports, or socializing with friends may be overwhelming for concussed adolescents suffering from headache, dizziness, nausea and fogginess, thus imposing heightened requirements on working memory to adequately function in such environments. While understanding the relationship between working memory and vestibular/ocular motor symptoms is critically important, no previous study has examined how an increase in working memory task difficulty affects the relationship between severity of vestibular/ocular motor symptoms and brain and behavioural responses in a working memory task. To address this question, we examined 80 adolescents (53 concussed, 27 non-concussed) using functional MRI while performing a 1-back (easy) and 2-back (difficult) working memory tasks with angry, happy, neutral and sad face distractors. Concussed adolescents completed the vestibular/ocular motor screening and were scanned within 10 days of injury. We found that all participants showed lower accuracy and slower reaction time on difficult (2-back) versus easy (1-back) tasks (*P*-values < 0.05). Concussed adolescents were significantly slower than controls across all conditions (*P* < 0.05). In concussed adolescents, higher vestibular/ocular motor screening total scores were associated with significantly greater differences in reaction time between 1-back and 2-back across all distractor conditions and significantly greater differences in retrosplenial cortex activation for the 1-back versus 2-back condition with neutral face distractors (*P*-values < 0.05). Our findings suggest that processing of emotionally ambiguous information (e.g. neutral faces) additionally increases the task difficulty for concussed adolescents. Post-concussion vestibular/ocular motor symptoms may reduce the ability to inhibit emotionally ambiguous information during working memory tasks, potentially affecting cognitive, academic and social functioning in concussed adolescents.

## Teaser

Manelis *et al.* report that post-concussion vestibular/ocular symptoms are associated with task difficulty-related decreases in retrosplenial cortex activation during a working memory task. Concussion disrupts the balance between activation and deactivation within the working memory circuitry, thus, potentially, leading to neurocognitive resource depletion in difficult cognitive tasks

## Graphical Abstract

**Graphical abstract:**
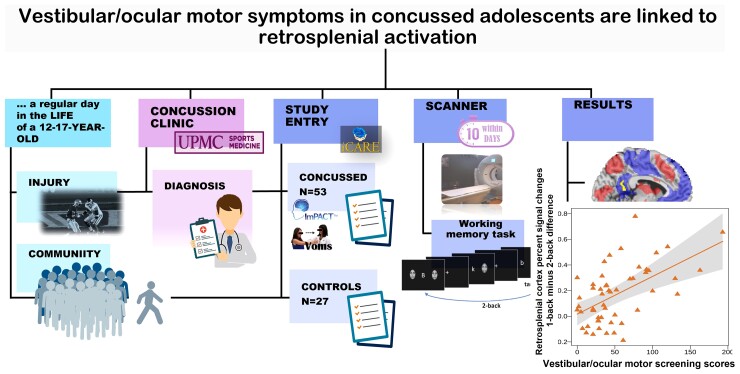

## Introduction

According to Center for Disease Control and Prevention, concussions leading to mild traumatic brain injuries in children and adolescents create a significant public health burden. Approximately 14% of adolescents between 12 and 17 years of age sustained one concussion during their lifetime, and over 5% adolescents sustain multiple concussions.^1^ Athletes have even higher odds of suffering from concussions.^2^ While the majority of concussed adolescents recover within the first 4 weeks, between 10 and 30% may experience persistent post-concussion symptoms lasting for more than 3 months.^3,4^ It has been suggested that post-concussion recovery of brain function might be delayed relative to recovery of performance on cognitive tasks and that functional MRI (fMRI) may provide means for evaluating recovery.^5,6^

Post-concussion symptoms include vestibular, ocular and motor symptoms that are associated with movement-related dizziness, blurry vision, unsteadiness and nausea.^7–9^ In addition to making movement more difficult, the vestibular/ocular motor symptoms result in headaches, fatigue, sleep dysregulation and create discomfort that might affect adolescents’ cognitive functioning, as well as their academic performance and social well-being.^10,11^ Experiencing vestibular/ocular motor symptoms during subacute post-concussion period predicts prolong recovery^12^ and represents an obstacle to engaging in a variety of activities when mental or physical exertion increases severity of these symptoms. Complex visual environments such as academic activities, playing sports or even socializing with friends may overwhelm concussed adolescents suffering from headache, dizziness, nausea and fogginess, thus requiring greater cognitive effort and greater involvement of cognitive resources.

Working memory supports on-line maintenance, manipulation and updating of information,^13^ and correlates with general intelligence,^14^ problem solving abilities^15^ and school performance.^16,17^ Working memory relies on the functioning of prefrontal, cingulate and posterior parietal cortical regions, whose activation increases when task difficulty increases, as well as default mode network (DMN) regions [e.g. posterior cingulate cortex (PCC) and retro-splenial cortex (RSC)], whose activation decreases with increasing levels of task difficulty.^13,18–21^ Considering that working memory circuitry undergoes developmental changes during adolescence^22–25^ and that damage to working memory brain regions may lead to a disruption of cognitive functioning,^18,26^ understanding the effects of concussion on working memory circuitry in adolescents is critically important, especially in the subacute post-concussion period.^27^

To date, neuroimaging research provides a range of findings that are often inconsistent across studies due to the use of particpants within a wide range of post-concussion periods (from several days to several months) and small sample sizes (often consisting of less than 20 participants per group). For example, in one study behavioural performance on a working memory task did not differ in concussed versus control adults, but the former showed significantly lower activation in the working memory circuitry during a more difficult task condition.^5^ In another study, concussed and control adults also did not differ from each other on *n*-back task performance; however, concussed participants showed significantly greater activation in the dorsolateral prefrontal cortex and parietal regions in a difficult versus easy working memory task condition.^6,28^ Still other studies reported worse behavioural performance and lower activation in prefrontal and parietal cortices during working memory tasks in concussed versus control adolescents both immediately after^29^ and within 3 months of an injury.^30^ A positive correlation between self-reported severity of post-concussion symptoms and the cerebellum activation for 2-back versus 0-back task was observed in concussed adolescents within a month of an injury.^31^

Concussion is associated with the alterations in the DMN^32^ possibly due to cerebrovascular changes that occur in acute/subacute periods of post-concussion recovery.^33^ Adolescents who had recovered from their concussion within 2 months showed greater activation in the ventral PCC and lower activation in the dorsal PCC during a working memory task, compared with those who still had post-concussive symptoms.^34^ Resting-state finctional connectivity in the DMN showed greater reduction in concussed adolescents with more severe post-concussion symptoms^35^ and after physical exertion.^36^

Despite the established relationship between working memory and oculomotor behaviour^37,38^ and known disruption of oculomotor behaviour caused by concussive injury,^39,40^ previous neuroimaging studies have not examined the relationship between the post-concussion vestibular/ocular motor symptoms and working memory in concussed adolescents. In this study, we aimed to fill this gap and examine the relationship between vestibular/ocular motor symptoms and behavioural and neural correlates of working memory in a large sample of concussed adolescents in the acute and subacute (1–10 days) post-concussion recovery period. Considering that concussions are often accompanied by emotion dysregulation,^41^ we wanted to test whether presenting emotional distractors with different emotion valences would differentially affect the relationship between vestibular/ocular motor symptoms and working memory in concussed adolescents. Working memory was examined using a modified version of the emotional faces *n*-back task^42^ in which angry, happy, neutral and sad faces were presented as distractors during an easy (1-back) and a difficult (2-back) working memory tasks. This paradigm allowed us to investigate the ability of concussed adolescents to inhibit irrelevant emotional (angry, happy or sad faces) and ambiguous (neutral faces) information in addition to their ability to cope with challenging working memory task. We hypothesized that inhibiting negative emotional distractors would be more difficult for concussed versus control adolescents due to known predisposition to experiencing depression symptoms in the former.^43^ It was proposed that increases in brain activation and extensive recruitment of prefrontal brain regions in concussed individuals may serve as a compensatory mechanism for a compromised abilty to maintain and process information in working memory.^29,44^ Based on this idea, and the previous findings described previously, we hypothesized that concussed adolescents with more severe vestibular/ocular motor symptoms will demonstrate greater differences in prefrontal, parietal and PCC regions during difficult versus easy working memory tasks than those with less severe vestibular/ocular motor sympoms and controls.

## Materials and methods

### Participants

The study was approved by the University of Pittsburgh Institutional Review Board. Written informed assent and consent were obtained from all participants and their parents. Participants included 70 adolescents with a concussion diagnosed within 10 days prior to scan and 30 adolescent healthy controls without history of concussions. All participants were between 12 and 17 years of age, right-handed, fluent in English and had IQ > 70. They also did not have contraindications for MRI (e.g. metal in the body, claustrophobia) and history of neurological, neurodevelopmental, or systemic medical illnesses, had no personal history of major psychiatric disorders or current (within the past 3 months) substance use/abuse. Current and prior history of concussions was an exclusion criterion for controls, but not concussed, adolescents in this study.

### Clinical and demographic assessments

Adolescents with suspected concussions were seen by a licensed healthcare professional trained in concussion (e.g. clinical neuropsychologist, physician) at the University of Pittsburgh Medical Center Concussion Clinic. Concussion was diagnosed using current consensus criteria^45^ within 0–8 days after injury. The information regarding history of previous concussions was collected using the Ohio State University Traumatic Brain Injury history form. Medical history, including history of headaches/migraines, was collected during clinical examination from concussed adolescents and their parents. Adolescents whose diagnosis of concussion was confirmed were offered to participate in the study. Concussed adolescents who agreed to participate were administered the vestibular/ocular motor screening (VOMS) and ImPACT concussion test^46^ by a clinician or trained clinical researcher from the study team on the same day as their visit to concussion clinic. The VOMS assesses symptom provocation following smooth pursuit, horizontal and vertical saccades, convergence, horizontal vestibular/ocular reflex, and visual motion sensitivity.^8^ Participants indicate the intensity of headache, dizziness, nausea, and fogginess on a scale from 0 (none) to 10 (severe) following each VOMS component. The scores from all scales were summed to compute a total VOMS score (range = 0–280). Given that all concussion assessments were administered at the concussion clinic, the concussion- and migraines-related information was collected from concussed adolescents only.

Information regarding participants’ age, biological sex, and IQ was collected from control and concussed participants. IQ was estimated using the Wechsler Abbreviated Scale of Intelligence that included the vocabulary and matrix reasoning subtests whose raw scores were adjusted by age, combined, and normalized. Research staff trained in administering psychiatric assessments evaluated history of major psychiatric disorders using the Mini International Neuropsychiatric Interview (M.I.N.I.) for children and adolescents,^47,48^ as well as the available medical records. The M.I.N.I. was administered to parent and child separately during the in-person visit preceding the MRI scanning session.

### Behavioral paradigm

**Figure 1:**
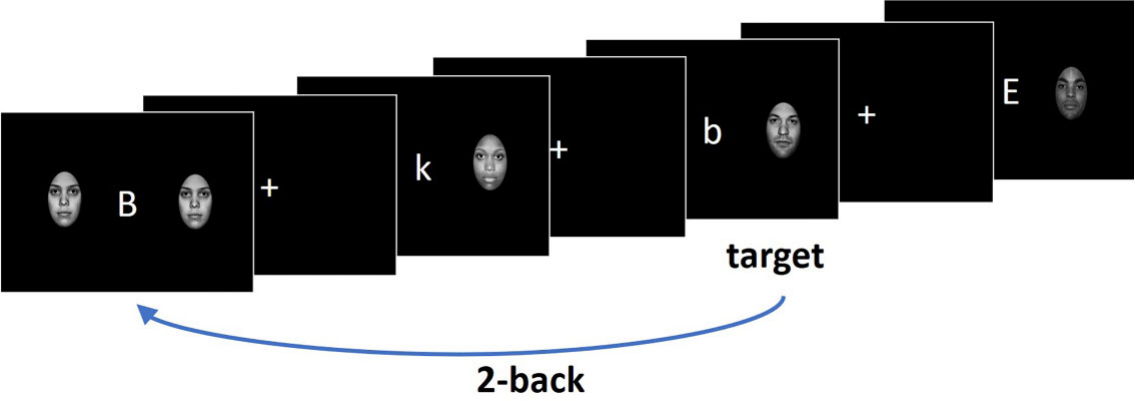
**The *n-*back task paradigm**.

The *n*-back task was modelled after the ef-nback task by Ladouceur.^42^ The modified task required participants to determine whether the letter presented on the screen matched the letter presented 1 (1-back) or 2 (2-back) trials earlier independent of whether the letter was capital or lowercase. The angry, happy, neutral and sad faces taken from the NimStim database^49^ were presented on the right and left sides of the letter and served as distractors (Fig. 1). Matching letters were called targets, and all other letters—non-targets. Participants performed three 5 min runs of the *n*-back task. Each run consisted of eight 30 s blocks of trials (1-back and 2-back tasks with four emotional face distractor conditions). A 4.5 s instruction screen was presented prior to each block of trials informing participants whether the upcoming block would be 1-back or 2-back. The blocks were separated with variable periods of rest that ranged from 5 to 15 s (mean = 10 s). To avoid systematic bias, the *n*-back sequences were generated for each participant individually using a Matlab script. Blocks were presented in random order. Each block consisted of 12 trials, of which five were targets. For each block, seven letters were randomly selected from a set of 19. Of these seven letters, one letter was presented three times (two times as a target), three letters were presented twice (one time as a target), and three other letters were presented once (never in the target position). For each emotional distractor condition, faces with relevant emotional expressions were randomly assigned to each trial in the block. The 500, 750 and 1000 ms inter-trial intervals were presented in the random order separating trials in the block. Participants were instructed to respond as quickly and accurately as possible within 2 s from trial onset. The responses were made with the index finger of one hand for targets and that of the other hand for non-targets. A hand assignment was counterbalanced across participants.

### Data analyses

#### Demographic and clinical variables

Demographic and clinical characteristics were compared between concussed and control adolescents using t-tests and chi-square tests (whichever was appropriate). A correlation analysis was used to examine the relationship between clinical and demographic variables. We used a Bonferroni correction to account for the number of correlation tests.

#### Behavioural measures

Mixed effects models with groups (concussed, control), task difficulty levels (1-back, 2-back), and distractor conditions (angry, happy, neutral, sad faces) as fixed factors, age and sex as covariates, and participant as random effect were used to examine interaction and main effects on reaction time (RT) for correct responses only and accuracy during the *n*-back task (‘lme4’,^50^ ‘lmerTest’,^51^ and ‘psycho’^52^ packages in R).

In concussed adolescents only, we used mixed effects models with task difficulty levels, distractor conditions, and either history of concussions or history of migraines as fixed factors to explore the effects of prior history of concussion or history of migraines/headaches on RT and accuracy. We also used a mixed effects model to examine the relationship between the VOMS total score × 2 task difficulty levels × 4 distractor conditions on RT and accuracy. In all models, participants were treated as random effect and participants’ age and sex were used as covariates.

#### Neuroimaging

##### Acquisition

Functional MRI data were acquired at the University of Pittsburgh using a Siemens PRISMA 3T MR system with a 32-channel RF head coil. A high-resolution structural image was acquired using the MPRAGE sequence (voxel size = 1 × 1 × 1 mm^3^, TR (repetition time) = 2400 ms, FOV = 256 mm, flip angle = 8°, 176 slices). Functional data (360 volumes per run) were collected using a gradient-echo, echo-planar sequence (voxel size: 2  ×  2 × 2 mm^3^, TR = 800 ms, echo time = 30 ms, field of view = 210 mm, flip angle = 52°, 72 slices, multiband acceleration factor = 8). Each participant completed three 5-min runs of the task. In addition, we collected two spin-echo images with the anterior-to-posterior and posterior-to-anterior phase-encoding directions (voxel size = 2  ×  2 × 2 mm^3^, TR = 8000 ms, TE = 66.00 ms, FOV = 210 mm, flip angle = 90°, 72 slices).

##### Preprocessing

The images were converted from DICOM to NIFTI using dcm2niix (v1.0.20180826 BETA GCC5.4.0).^53^ The optiBET script^54^ was used to remove non-brain tissues. Susceptibility distortions of BOLD images were estimated based on two spin-echo images collected in the anterior-to-posterior and poster-to-anterior phase-encoding directions using *topup*^55^ in FSL6.0.3 (www.fmrib.ox.ac.uk/fsl). Motion correction was performed using MCFLIRT^56^ and spatial smoothing with a Gaussian kernel of full-width at half-maximum = 6 mm was applied. To transform BOLD images to MNI space, BOLD images were first registered to the high-resolution structural (MPRAGE) images using FLIRT (FMRIB’s Linear Image Registration Tool)^56,57^ with BBR, the high-resolution images were registered to the MNI152_T1_2 mm template using FNIRT (FMRIB’s Non-linear Image Registration Tool)^58^ with nine degrees of freedom, and the two resulting transformations were concatenated and applied to the original BOLD. The quality of transformation was checked for every participant.

To remove motion artefacts, ICA-AROMA^59^ was applied to BOLD images that were preprocessed and registered to MNI space. The *fsl_anat* script (http://fsl.fmrib.ox.ac.uk/fsl/fslwiki/fsl_anat) was used to segment high-resolution structural images to white matter, grey matter, and CSF. The white matter and CSF masks were then co-registered with functional images, and their time courses were extracted from the preprocessed functional data for further analyses. These time courses, together with the first five TR, were regressed out from the preprocessed BOLD images. After that, a high-pass filter (Gaussian-weighted least-squares straight line fitting, with sigma = 56.25) was applied. No slice-timing correction was applied.

##### First-level analysis

Preprocessed data were submitted to a first-level General Linear Model analysis implemented using FEAT (FMRI Expert Analysis Tool, v6.0). A haemodynamic response function was modelled using a Gamma function. The model included eight regressors (happy, angry, fear and sad face distractor conditions that were presented in 1-back and 2-back task difficulty conditions). The contrasts of interest included computing the 1-back versus 2-back differences in brain activation across all face distractors, as well as for each face distractor separately.

##### Group-level analyses

A working memory circuitry was identified as the set of brain regions that showed changes in activation for 1-back versus 2-back tasks across all participants and all distractor conditions. For this analysis, we used the Sandwich Estimator (*swe*) approach^60^ for nonparametric permutation inference conducted in the whole brain with 5000 permutations and a cluster-level correction of *z* = 3.1 and *P* = 0.025 (=0.05/2) to apply Bonferroni correction for the two contrasts of interest (1-back > 2-back and 2-back > 1-back). The activation maps for these two contrasts of interest were then combined into a single binary mask.

This mask was used to investigate the relationship between the VOMS scores and brain activation associated with the changes in the task difficulty (i.e. 1-back versus 2-back) in concussed adolescents only. For this analysis, we used the *swe* analyses conducted in the working memory circuitry mask with 5000 permutations and a cluster-level correction of *z* = 3.1 and *p*-corrected = 0.00625 (0.05/8) to apply Bonferroni correction for two directions of the effect (a positive and a negative correlation with VOMS scores) and four distractors (angry, happy, neutral and sad).

To interpret the findings in concussed adolescents, we first used *featquery* to extract BOLD percent signal changes from the regions showing the relationship with VOMS. Then, we computed the 1-back minus 2-back activation differences in all participants. Then, we conducted a *k*-means cluster analysis in concussed adolescents only using the 1-back-minus-2-back differences in RSC activation as an input variable. To obtain groups of reasonable sizes, we have chosen *k* = 2. The VOMS scores, as well as age and sex composition were compared between the two resulting clusters. In addition, we conducted a one-way ANOVA with the 1-back-minus-2-back differences in RSC activation as an outcome variable and group (controls, concussed Cluster 1 and concussed Cluster 2) as a predictor variable. *Post hoc* between-group contrasts were estimated using the *estimate_contrasts* function in ‘modelbased’ package in R with FDR correction for multiple comparisons.

##### Exploratory analyses

To rule out the contribution of previous concussions and history of migraine on our main findings, we conducted mixed effects analyses in concussed adolescents with two group (history/no history of concussion)-by-nback-by-4 distractor conditions on the BOLD percent signal changes in the region(s) identified in the main analyses.

### Data availability

The data will be available upon reasonable request.

## Results

### Demographic and clinical variables

Seventeen of 70 concussed (24.3%) and three of 30 control (10%) adolescents were excluded from the analysis due to incomplete neuroimaging acquisition [8 (11.4%) concussed, two (6.7%) control), excessive motion >4 mm in any direction (5 (7.1%) concussed, one (3.3%) controls], poor behavioural performance [two (2.9%) concussed], or poor image quality [two (2.9%) concussed], thus leaving 53 concussed and 27 control adolescents in the analyses. Among concussed adolescents included into the analyses, 15 reported prior history of concussion and 17 reported a history of migraines. Sports participation among adolescents included into the analyses was 96.2% for concussed adolescents and 96.3% for control adolescents. Forty-six (86.8%) adolescents were concussed due to sport-related injuries, one (1.9%) due to a car accident, five (9.4%) due to falling or having other accidents, and one (1.9%) due to other reasons. Concussed and control groups of participants did not differ from each other in age, IQ, or gender composition (Table 1).

**Table 1:** Demographic and clinical characteristics

	Concussed	Controls	Statistics
N	53	27	
Number of females (%)	20 (38%)	14 (52%)	χ^2^ (1) = 0.94, *P* = 0.33
Race			χ^2^ (2) = 0.68, *P* = 0.71
• Number of Caucasian (%)	• 45 (85%)	• 21 (78%)	
• Number of Black (%)	• 7 (13%)	• 5 (19%)	
• Number of unknown (%)	• 0	• 1 (4%)	
Mean age in years (SD)	15.58 (1.57)	15.31 (1.38)	t(78) = 0.78, *P* = 0.43
(min-max)	(12.1–17.9)	(12.7–17.4)	
Mean IQ (SD)	104.3 (8.4)	107.9 (8.0)	t(77) = −1.8, *P* = 0.07
(min, max)	(83.0–122.0)	(92.0–124.0)	
Mean VOMS scores total (SD)	51.77(42)	—	na
(min, max)	(0.0–193.0)		
Mean ImPACT PCSS scores total (SD)	28.6 (19.8)	—	na
(min, max)	(0.0–79.0)		
Mean number of days between injury and clinical assessment (SD)	3.6 (1.7)	—	na
(min, max)	(0–8)		
Mean number of days between injury and scan (SD)	7 (2.4)	—	na
(min, max)	(1–10)		
Number of participants with previous history of concussion (%)	15 (28%)	—	na
Number of participants with history of migraines (%)	17 (32%)	—	na

Note: na, not applicable; SE, standard errors

In concussed adolescents, the VOMS scores positively correlated with the ImPACT PCSS scores (*r* = 0.61, *P* < 0.001). However, the VOMS and ImPACT PCSS scores did not depend on prior history of concussions (VOMS: *t*(51) = −0.3, *P* = 0.75; ImPACT PCSS: *t*(51) = 0.8, *P* = 0.42) or history of migraines/headaches (VOMS: *t*(51) = −1.34, *P* = 0.19; ImPACT PCSS: *t*(51) = −1.35, *P* = 0.18). Neither VOMS nor ImPACT PCSS significantly correlated with the number of days between injury and clinical assessment (VOMS: *r* = 0.07, *P* = 0.62; ImPACT PCSS: *r* = 0.06, *P* = 0.65), the number of days between clinical assessment and scan (VOMS: *r* = −0.06, *P* = 0.68; ImPACT PCSS: *r* = 0.09, *P* = 0.54), age (VOMS: *r* = 0.22, *P* = 0.2; ImPACT PCSS: *r* = 0.00, *P* = 0.99), or IQ (VOMS: *r* = 0.08, *P* = 0.58; ImPACT PCSS: *r* = −0.07, *P* = 0.6).

Considering that about 25% of concussed adolescents were excluded from the neuroimaging data analysis, we wanted to ensure that the distributions of included and excluded concussed adolescents are similar. Therefore, we compared the VOMS and ImPACT PCSS scores, age, IQ, and sex composition in included versus excluded concussed adolescents using *t*-tests and χ^2^ test as appropriate. Three excluded concussed adolescents had missing VOMS and ImPACT PCSS values. One included and one excluded concussed adolescents had missing IQ values. Excluded participants were signficantly younger than included participants (mean[SD] excluded = 14.2[1.4]; mean[SD] included = 15.6[1.6]; *t*(68) = −3.2, *P* = 0.002), but did not differ in IQ [mean (SD) excluded = 104.4 (9.2); mean (SD) included = 104.3 (8.4); *t*(66) = 0.02, *P* = 0.98], sex composition (*P*-values > 0.05), VOMS scores [mean (SD) excluded (38.3): 57.7, mean (SD) included = 51.8 (42); *t*(65) = 0.48, *P*-value = 0.63], and ImPACT PCSS scores [mean (SD) excluded: 37.4 (16.5), mean (SD) included = 29.6 (19.7); *t*(65) = 1.5, *P*-value = 0.13].

### Behavioural measures

**Figure 2:**
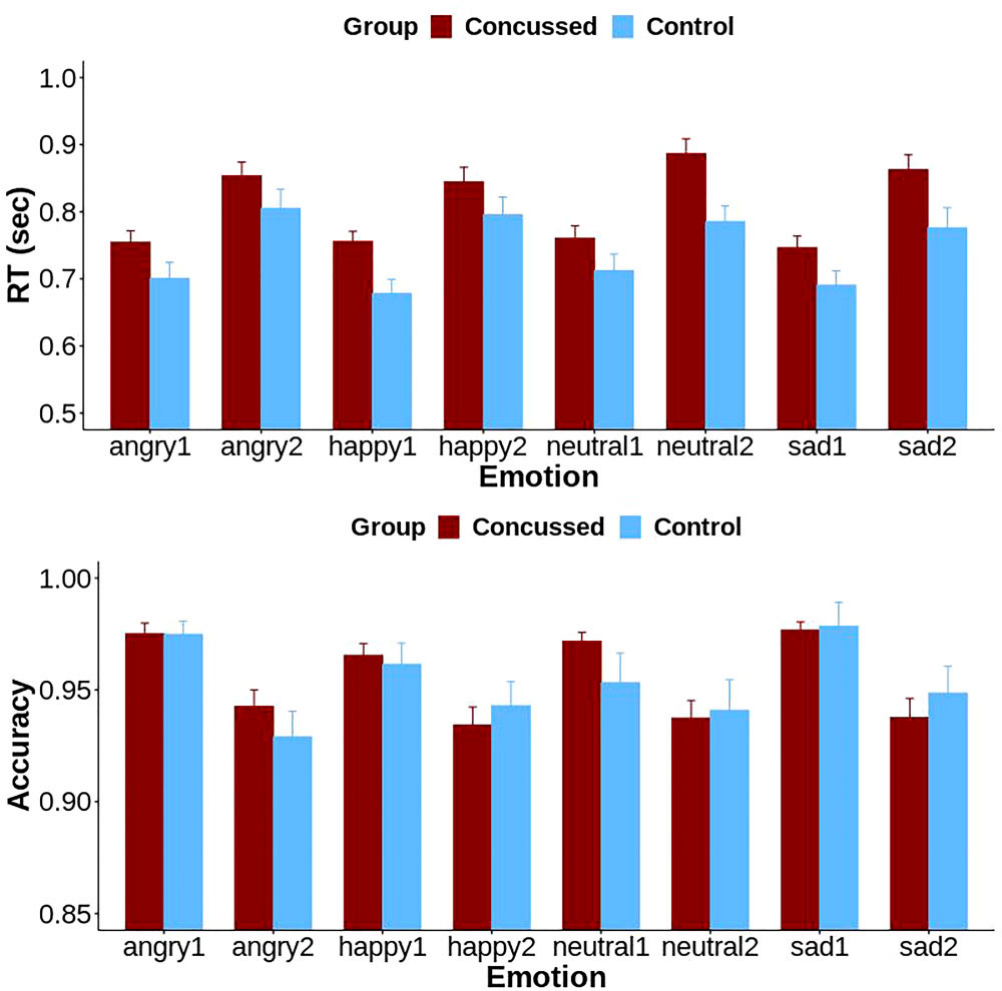
**RT and accuracy in the 1-back and 2-back task difficulty conditions with angry, happy, neutral and sad face distractors in concussed adolescents and controls**. Mixed effects models showed a significant main effect of task difficulty on RT (*F*(1,546) = 258.1, *P* < 0.001) and accuracy (*F*(1,546) = 87.7, *P* < 0.001). Participants were significantly slower and less accurate for the 2-back versus 1-back task difficulty condition [RT: *t*(546) = 16.1, *P* < 0.001; accuracy: *t*(546) = −9.36, *P* < 0.001]. In addition, there was a main effect of group on RT (*F*(1,76) = 6.4, *P* = 0.013), with concussed adolescents being significantly slower than controls (*t*(76) = 2.53, *P* = 0.014).

In all 80 participants, the two mixed effects models (one for RT and one for accuracy) revealed a significant main effect of task difficulty (i.e. 1-back versus 2-back) on RT (*F*(1,546) = 258.1, *P* < 0.001) and accuracy (*F*(1,546) = 87.7, *P* < 0.001), with participants being significantly slower and less accurate for the 2-back versus 1-back task difficulty condition (RT: *t*(546) = 16.1, *P* < 0.001; accuracy: *t*(546) = −9.36, *P* < 0.001). In addition, there was a main effect of group on RT (*F*(1,76) = 6.4, *P* = 0.013), with concussed adolescents being significantly slower than controls (*t*(76) = 2.53, *P* = 0.014). There was no significant effect of group on accuracy nor a main effect of distractor, sex, age or any interaction effects on RT and accuracy (Fig. 2).

**Figure 3:**
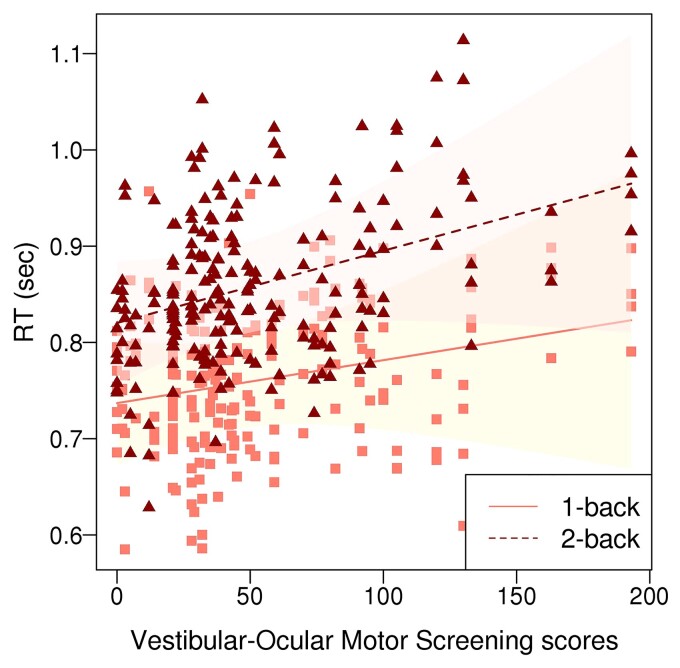
**The *n*-back by VOMS scores interaction effect on RT in concussed adolescents**. The mixed effect model showed a significant effect of task difficulty by VOMS interaction on RT (*F*(1,357) = 4.45, *P* = 0.036) with greater RT differences for 2-back versus 1-back conditions observed in concussed adolescents with more severe vestibular/ocular motor symptoms across all distractors.

In the 53 concussed adolescents only, the mixed effect model testing the effect of task difficulty-by-distractor-by-VOMS interaction on RT and accuracy revealed a significant effect of task difficulty by VOMS interaction on RT (*F*(1,357) = 4.45, *P* = 0.036) with greater RT differences for 2-back versus 1-back conditions observed in concussed adolescents with more severe vestibular/ocular motor symptoms across all distractors (Fig. 3). There were no significant difficulty-by-distractor-by-VOMS interaction effects on RT or accuracy, as well as no other main or interaction effects on accuracy.

The two additional mixed effect models (one for history of concussion, one for history of migraines/headache) revealed no significant effect of previous history of concussions nor migraine/headache on RT and accuracy in concussed adolescents.

### Neuroimaging

**Figure 4:**
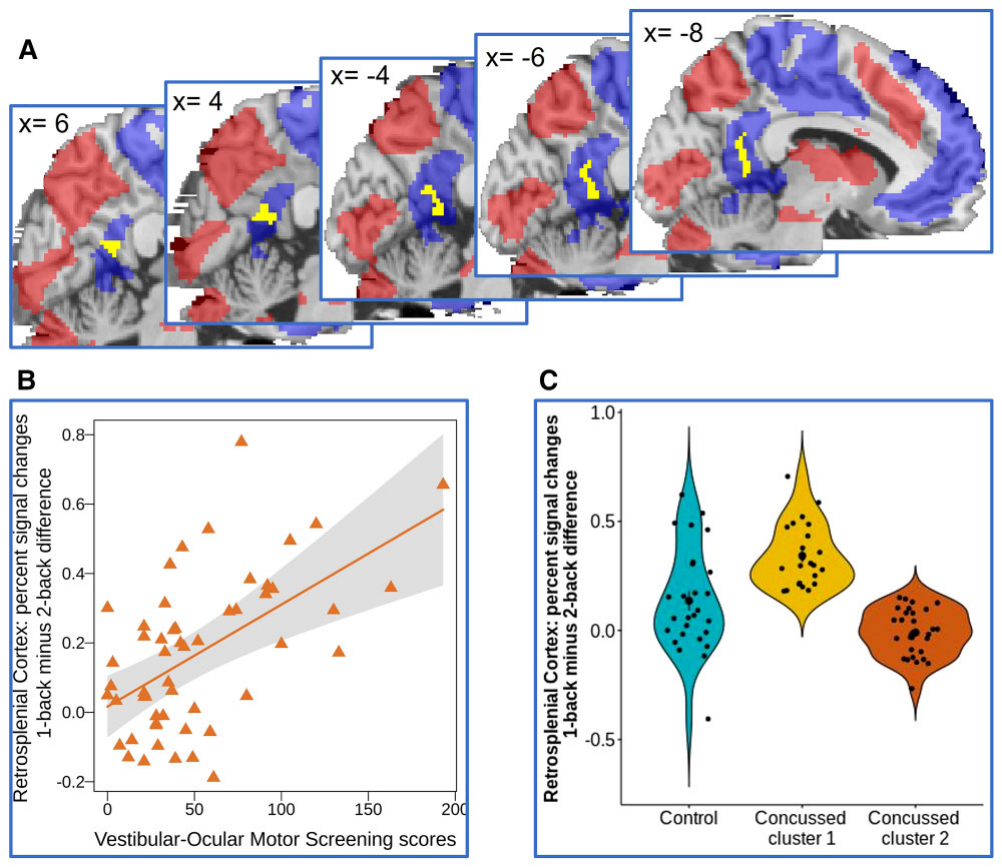
**The relationship between VOMS scores and task difficulty-related changes (1-back versus 2-back) in the retrosplenial cortex (RSC) in concussed adolescents**. (**A**) The RSC region (in yellow) whose 1-back minus 2-back activation differences significantly correlated with the VOMS scores. This activation cluster is plotted over the working memory circuitry mask (1-back > 2-back is in blue, and 2-back > 1-back is in red). (**B**) The scatter plot illustrating the relationship between the VOMS scores and 1-back minus 2-back activation differences in the RSC in concussed adolescents (*P* = 0.0032). The confidence band with the alpha level = 0.05 is shown in grey. (**C**) The 1-back minus 2-back RSC activation differences in healthy controls and concussed adolescents from clusters 1 and 2 (per the k-mean cluster analysis). A one-way ANOVA revealed a significant effect of group (F(2,77) = 27.7, *P* < 0.0001) on the 1-back-minus-2-back differences in RSC activation. Control adolescents had significantly lower 1-back-minus-2-back differences in RSC activation than concussed adolescents in Cluster 1 (t(77) = −4.26, p-fdr-corrected < 0.001) but significantly greater differences than those in concussed adolescents in Cluster 2 (t(77) = 3.22, p-fdr-corrected = 0.002). Concussed adolescents in Cluster 1 had significantly greater differences in RSC activation than those in Cluster 2 (t(77)7.44, p-fdr-corrected < 0.001).

The working memory circuitry regions determined across all 80 adolescents (Fig. 4A: 2-back > 1-back in red and 1-back > 2-back in blue) were consistent with previous findings^18^ and are reported in Supplementary Table 1 and Supplementary Table 2.

In the 53 concussed adolescents only, the neuroimaging data analysis conducted in the working memory circuitry revealed a cluster of voxels located in the bilateral RSC [*P* = 0.0032, *nvox* = 103, (−8, −52, 18), BA29 and 30; Fig. 4A]. The 1-back versus 2-back activation differences in the RSC in the neutral face distractor condition were greater for concussed individuals with more severe vestibular/ocular motor symptoms (higher VOMS scores) than for those with less severe symptoms (Fig. 4B). These activation differences did not correlate with the 1-back versus 2-back changes in RT (*r* = −0.02; *P* = 0.9) or accuracy (*r* = −0.25, *P* = 0.07). No significant relationships between the VOMS scores and differential RSC activation were found for happy, angry or sad face distractor conditions.

The *k*-means cluster analysis with *k* = 2 has identified clusters of 23 (Cluster 1) and 30 concussed adolescents (Cluster 2). Concussed adolescents in Cluster 1 had significantly higher VOMS score than concussed adolescents in Cluster 2 [Cluster 1 mean (SD) = 73.34 (46.6); cluster 2 mean (SD) = 35.2 (29.3); *t*(51) = 3.6, *P* = 0.0006] but did not significantly differ in age (*t*(51) = 1.3, *P* = 0.2), IQ (*t*(48) = 0.57, *P* = 0.57), or sex composition (χ^2^ (1) = 0.03, *P* = 0.85). A one-way ANOVA revealed a significant effect of group (*F*(2,77) = 27.7, *P* < 0.0001) on the 1-back-minus-2-back differences in RSC activation (Fig. 4C). A *post hoc* analysis of contrasts showed that control adolescents had significantly lower 1-back-minus-2-back differences in RSC activation than concussed adolescents in Cluster 1 (*t*(77) = −4.26, *p-fdr-corrected* < 0.001) but significantly greater differences than those in concussed adolescents in Cluster 2 (*t*(77) = 3.22, *p-fdr-corrected* = 0.002). Concussed adolescents in Cluster 1 had significantly greater differences in RSC activation than those in Cluster 2 (t(77)7.44, *p-fdr-corrected* < 0.001).

### Exploratory analyses

Of 53 concussed adolescents, 15 reported prior history of concussions and 17 reported history of migraines. The mixed effects analyses that compared 38 concussed adolescents without prior history of concussions and 15 concussed adolescents with prior history of concussions revealed a significant concussion history-by-task difficulty interaction effect on the 1-back versus 2-back activation differences in the RSC for neutral faces (*F*(1,350) = 4.2, *P* = 0.04), with greater changes in activation from 1-back to 2-back condition in concussed adolescents with a previous history of concussions. There was no significant main or interaction effects on history of migraines on RSC activation.

## Discussion

Vestibular/ocular motor post-concussion symptoms affect every-day functioning and may predict slower recovery in concussed individuals.^7,12^ In this study, we examined for the first time the relationship between vestibular/ocular motor symptoms in a subacute phase of concussion and behavioral and brain responses to increasing task difficulty in adolescents who sustained a recent concussion. Concussed adolescents were slower than healthy controls to respond in the *n*-back task but did not differ from controls in the magnitude of the 1-back versus 2-back differences in RT and accuracy, which was consistent with previous studies.^5,6^ Concussed adolescents with more severe VOMS symptoms showed a greater slowing in RTs, independent of face distractors and greater deactivation in the bilateral RSC for a more difficult 2-back task versus easier 1-back task in the context of emotionally neutral face distractors. Our findings linking vestibular/ocular motor symptoms to disrupted functioning of the RSC in response to increased task difficulty may help explain worsening of concussion symptoms during mental exertion in concussed adolescents.

The RSC (BA 29 and BA 30) is a part of the posterior cingulum cortex and DMN that decreases in activation during performance on more difficult versus easier working memory tasks.^13,18–21^ The RSC is reciprocally connected to the hippocampal formation, anterior thalamus, PCC, DLPFC, frontopolar PFC and anterior cingulate cortex.^61^ It is sometimes considered to be a critical node mediating “the related functions of spatial cognition, context representation and episodic memory”.^62(p11)^ Damage to the RSC affects memory retrieval ^63–65^, as well as autobiographical^66^ and emotional^67^ memory. Metabolic reduction in the RSC contribute to mild cognitive impairment^68,69^ and amyloid formation,^70^ which plays a role in the early stages of Alzheimer’s disease.^61^

Despite apparent connections between RSC function and neurological conditions,^61,70^ the RCS’s role during subacute post-concussion recovery has not been examined in general and in the context of working memory tasks specifically. Even though one prior study showed the RSC involvement in spatial working memory in rats,^71^ no study characterized the role of the RSC in human working memory. Our study highlighted the role of the RSC in concussed adolescents when performing a working memory task with neutral face distractors. Neutral faces are ambiguous in their emotional content and may be misclassified as emotional faces,^72^ likely creating additional cognitive difficulties for concussed adolescents, especially when completing more challenging tasks. This RSC’s effect in the *n*-back task with neutral face distractors is consistent with previous findings of RSC involvement in tasks that elicit strong contextual associations^62,73,74^ as well as updating spatial information.^75^ Our findings showing that adolescents with severe vestibular/ocular motor post-concussion symptoms were slower and had greater task difficulty-related decreases in the RSC than those with less severe symptoms, indicating that disfunction of the vestibular, ocular, and motor systems may affect the ability of concussed adolescents to process emotionally ambiguous information in tasks that require significant mental effort. Future studies should consider using emotional stimuli rather than emotional distractors to examine how working memory concussed adolescents is linked to the relationship between the vestibular/ocular motor symptoms and emotionally salient and emotionally ambiguous information.

It is noteworthy that concussed adolescents with prior history of concussion had greater deactivation in the RSC as compared with those with no such history, as well as healthy controls. These findings suggest that the working memory circuitry can flexibly adjust to accommodate clinical severity to ensure adequate task performance. Different neurobiological mechanisms may be activated depending on the severity of vestibular/ocular motor symptoms. For example, more symptomatic adolescents who had significantly greater RSC deactivation than controls in the difficult task may use a compensatory mechanism to help maintain and process information in working memory.^29,44^ However, less symptomatic adolescents who deactivated the RSC significantly less than controls may re-distribute working memory resources to decrease the DMN involvement in the task. The differences in activation patterns observed between more symptomatic and less symptomatic concussed adolescents may explain the discrepancies in the previous concussion-related neuroimaging findings. Recent studies suggest that the DMN connectivity, as well as the connectivity between the PCC and other brain regions, becomes stronger during child development.^76^ Based on these findings, we hypothesize that disruption of one of the main nodes in the DMN during brain development might affect the future DMN development after concussion. Future longitudinal neuroimaging studies of posts-concussion recovery should test this hypothesis.

The RSC’s role in working memory during post-concussion recovery warrants further investigation due to several limitations of this study. One limitation is the cross-sectional design. Future studies should investigate how vestibular/ocular motor symptoms improvement over time is related to dynamic changes in task difficulty-related RSC activation over time during post-concussion recovery. The other limitation of our study is a lack of clinical data regarding history of migraines and the VOMS scores in control adolescents, which did not allow us to compare the effects VOMS and migraine in concussed and control individuals. Future studies should collect this information across all individuals in the study. Although our study has a large and homogeneous (in terms of a recovery period) sample of concussed adolescents, it would be beneficial to combine samples from multiple centres to ensure better results generalizability.

In summary, we found that the severity of vestibular/ocular motor symptoms was related to the pattern of the RSC response to increases in *n*-back task difficulty: concussed adolescents with greater difficulty-related decreases in the RSC than controls had more severe vestibular/ocular motor symptoms that their peers with lower difficulty-related decreases in the RSC than controls. Considering that vestibular/ocular motor symptoms were related to activation in the DMN rather that activation in the regions showing task difficulty-related increases, we propose that concussion disrupts the balance between activation and deactivation within the working memory circuitry, thus, potentially, leading to neurocognitive resource depletion in difficult cognitive tasks. This effect might be more pronounced in the context of ambiguous emotional stimuli (e.g. neutral faces) that may be difficult to ignore when they appear as distractors and whose processing may require more neurocognitive resources than processing of salient stimuli.

## Supplementary Material

fcac123_Supplementary_Data

## Abbreviations

DMN =: default mode network
PCC =: posterior singulate cortex
RSC =: retrosplenial cortex
RT =: reaction time
VOMS =: vestibular/ocular motor screening

## References

[1] Veliz P , McCabe SE, Eckner JT, Schulenberg JE. Prevalence of concussion among US adolescents and correlated factors. JAMA. 2017;318:1180–1182. 28973604 10.1001/jama.2017.9087 PMC5817894

[2] Veliz P , Eckner JT, Zdroik J, Schulenberg JE. Lifetime prevalence of self-reported concussion among adolescents involved in competitive sports: A national US study. J Adolesc Heal. 2019;64:272–275. 10.1016/j.jadohealth.2018.08.023 PMC6339843 30409755

[3] Yeates KO , Kaizar E, Rusin J, et al Reliable change in postconcussive symptoms and its functional consequences among children with mild traumatic brain injury. Arch Pediatr Adolesc Med. 2012;166:615–622. 22393171 10.1001/archpediatrics.2011.1082 PMC3537865

[4] Babcock L , Byczkowski T, Wade SL, Ho M, Mookerjee S, Bazarian JJ. Predicting postconcussion syndrome after mild traumatic brain injury in children and adolescents who present to the emergency department. JAMA Pediatr. 2013;167:156–161. 23247384 10.1001/jamapediatrics.2013.434 PMC4461429

[5] Chen CJ , Wu CH, Liao YP, et al Working memory in patients with mild traumatic brain injury: Functional MR imaging analysis. Radiology. 2012;264:844–851. 22829681 10.1148/radiol.12112154

[6] Dettwiler A , Murugavel M, Putukian M, Cubon V, Furtado J, Osherson D. Persistent differences in patterns of brain activation after sports-related concussion: A longitudinal functional magnetic resonance imaging study. J Neurotrauma. 2014;31:180–188. 23914845 10.1089/neu.2013.2983 PMC3900041

[7] Wallace B , Lifshitz J. Traumatic brain injury and vestibulo-ocular function: Current challenges and future prospects. Eye Brain. 2016;8:153–164. 28539811 10.2147/EB.S82670 PMC5398755

[8] Mucha A , Collins MW, Elbin RJ, et al A brief vestibular/ocular motor screening (VOMS) assessment to evaluate concussions: Preliminary findings. Am J Sports Med. 2014;42:2479–2486. 25106780 10.1177/0363546514543775 PMC4209316

[9] Kontos AP , Deitrick JMA, Collins MW, Mucha A. Review of vestibular and oculomotor screening and concussion rehabilitation. J Athl Train. 2017;52:256–261. 28387548 10.4085/1062-6050-51.11.05 PMC5384823

[10] Barlow KM , Crawford S, Stevenson A, Sandhu SS, Belanger F, Dewey D. Epidemiology of postconcussion syndrome in pediatric mild traumatic brain injury. Pediatrics. 2010;126:e374–e381. 20660554 10.1542/peds.2009-0925

[11] Blume H , Hawash K. Subacute concussion-related symptoms and postconcussion syndrome in pediatrics. Curr Opin Pediatr. 2012;24:724–730. 23128838 10.1097/MOP.0b013e328359e4cc

[12] Kontos AP , Elbin RJ, Schatz P, et al A revised factor structure for the post-concussion symptom scale: Baseline and postconcussion factors. Am J Sports Med. 2012;40:2375–2384. 22904209 10.1177/0363546512455400

[13] Baddeley A . Working memory. Curr Biol. 2010;20:R136–R140. 20178752 10.1016/j.cub.2009.12.014

[14] Conway ARA , Kane MJ, Engle RW. Working memory capacity and its relation to general intelligence. Trends Cogn Sci. 2003;7:547–552. 14643371 10.1016/j.tics.2003.10.005

[15] Chuderski A , Jastrzebski J. Much ado about aha! : Insight problem solving is strongly related to working memory capacity and reasoning ability. J Exp Psychol Gen. 2018;147:257–281. 29058940 10.1037/xge0000378

[16] Swanson HL . Working memory, attention and mathematical problem solving: A longitudinal study of elementary school children. J Educ Psychol. 2011;103:821–837.

[17] Alloway TP . How does working memory work in the classroom? Educ Res Rev 2006;1:134–139.

[18] Owen AM , McMillan KM, Laird AR, Bullmore E. N-back working memory paradigm: A meta-analysis of normative functional neuroimaging studies. Human Brain Mapping. 2005;25:46–59. 15846822 10.1002/hbm.20131 PMC6871745

[19] Manelis A , Reder LM. He who is well prepared has half won the battle: An fMRI study of task preparation. Cereb Cortex. 2015;25:726–735. 24092642 10.1093/cercor/bht262 PMC4318533

[20] Manelis A , Reder LM. Effective connectivity among the working memory regions during preparation for and during performance of the n-back task. Front Hum Neurosci. 2014;8:593. 25140143 10.3389/fnhum.2014.00593 PMC4122182

[21] Manelis A , Iyengar S, Swartz HA, Phillips ML. Prefrontal cortical activation during working memory task anticipation contributes to discrimination between bipolar and unipolar depression. Neuropsychopharmacology. 2020;45:956–963. 32069475 10.1038/s41386-020-0638-7 PMC7162920

[22] Schweinsburg AD , Nagel BJ, Tapert SF. fMRI reveals alteration of spatial working memory networks across adolescence. J Int Neuropsychol Soc. 2005;11:631–644. 16212691 10.1017/S1355617705050757 PMC2270702

[23] Schleepen TMJ , Jonkman LM. The development of non-spatial working memory capacity during childhood and adolescence and the role of interference control: An N-back task study. Dev Neuropsychol. 2010;35(1):37–56. 20390591 10.1080/87565640903325733

[24] Geier CF , Garver K, Terwilliger R, Luna B. Development of working memory maintenance. J Neurophysiol. 2009;101:84–99. 18971297 10.1152/jn.90562.2008 PMC2637004

[25] Yaple Z , Arsalidou M. N-back working memory task: Meta-analysis of normative fMRI studies with children. Child Dev. 2018;89:2010–2022. 29732553 10.1111/cdev.13080

[26] Curtis CE , D’Esposito M. The effects of prefrontal lesions on working memory performance and theory. Cogn Affect Behav Neurosci. 2004;4:528–539. 15849895 10.3758/cabn.4.4.528

[27] Henry LC , Elbin RJ, Collins MW, Marchetti G, Kontos AP. Examining recovery trajectories after sport-related concussion with a multimodal clinical assessment approach. Neurosurgery. 2016;78:232–241. 26445375 10.1227/NEU.0000000000001041 PMC4833014

[28] McAllister TW , Saykin AJ, Flashman LA, et al Brain activation during working memory 1 month after mild traumatic brain injury: A functional MRI study. Neurology. 1999;53:1300–1308. 10522888 10.1212/wnl.53.6.1300

[29] Hammeke TA , McCrea M, Coats SM, et al Acute and subacute changes in neural activation during the recovery from sport-related concussion. J Int Neuropsychol Soc. 2013;19:863–872. 23829951 10.1017/S1355617713000702

[30] Keightley ML , Singh Saluja R, Chen JK, et al A functional magnetic resonance imaging study of working memory in youth after sports-related concussion: Is it still working? J Neurotrauma. 2014;31:437–451. 24070614 10.1089/neu.2013.3052 PMC3934544

[31] Krivitzky LS , Roebuck-Spencer TM, Roth RM, Blackstone K, Johnson CP, Gioia G. Functional magnetic resonance imaging of working memory and response inhibition in children with mild traumatic brain injury. J Int Neuropsychol Soc. 2011;17:1143–1152. 22014100 10.1017/S1355617711001226

[32] Johnson B , Zhang K, Gay M, et al Alteration of brain default network in subacute phase of injury in concussed individuals: Resting-state fMRI study. Neuroimage. 2012;59:511–518. 21846504 10.1016/j.neuroimage.2011.07.081 PMC3196274

[33] Militana AR , Donahue MJ, Sills AK, et al Alterations in default-mode network connectivity may be influenced by cerebrovascular changes within 1 week of sports related concussion in college varsity athletes: A pilot study. Brain Imaging Behav. 2016;10:559–568. 25972119 10.1007/s11682-015-9407-3 PMC4644725

[34] Stein A , Iyer KK, Khetani AM, Barlow KM. Changes in working memory-related cortical responses following pediatric mild traumatic brain injury: A longitudinal fMRI study. J Concussion. 2021;5:1–14.

[35] Iyer KK , Zalesky A, Barlow KM, Cocchi L. Default mode network anatomy and function is linked to pediatric concussion recovery. Ann Clin Transl Neurol. 2019;6:2544–2554. 31755665 10.1002/acn3.50951 PMC6917315

[36] Zhang K , Johnson B, Gay M, et al Default mode network in concussed individuals in response to the YMCA physical stress test. J Neurotrauma. 2012;29:756–765. 22040294 10.1089/neu.2011.2125 PMC3303100

[37] Walter K , Bex P. Cognitive load influences oculomotor behavior in natural scenes. Sci Rep. 2021;11:1–12. 34117336 10.1038/s41598-021-91845-5 PMC8196072

[38] Van der Stigchel S . The search for oculomotor inhibition interactions with working memory. Exp Psychol. 2010;57:429–435. 20178939 10.1027/1618-3169/a000053

[39] Capó-Aponte JE , Urosevich TG, Temme LA, Tarbett AK, Sanghera NK. Visual dysfunctions and symptoms during the subacute stage of blast-induced mild traumatic brain injury. Mil Med. 2012;177:804–813. 22808887 10.7205/milmed-d-12-00061

[40] Heitger MH , Jones RD, MacLeod AD, Snell DL, Frampton CM, Anderson TJ. Impaired eye movements in post-concussion syndrome indicate suboptimal brain function beyond the influence of depression, malingering or intellectual ability. Brain. 2009;132:2850–2870. 19617197 10.1093/brain/awp181

[41] Christensen J , Eyolfson E, Salberg S, Mychasiuk R. Traumatic brain injury in adolescence: A review of the neurobiological and behavioural underpinnings and outcomes. Dev Rev. 2021;59:100943.

[42] Ladouceur CD , Silk JS, Dahl RE, Ostapenko L, Kronhaus DM, Phillips ML. Fearful faces influence attentional control processes in anxious youth and adults. Emotion. 2009;9:855–864. 20001128 10.1037/a0017747

[43] Chrisman SPD , Richardson LP. Prevalence of diagnosed depression in adolescents with history of concussion. J Adolesc Heal. 2014;54:582–586. 10.1016/j.jadohealth.2013.10.006 PMC3999295 24355628

[44] Newsome MR , Steinberg JL, Scheibel RS, et al Effects of traumatic brain injury on working memory-related brain activation in adolescents. Neuropsychology. 2008;22:419–425. 18590353 10.1037/0894-4105.22.4.419

[45] McCrory P , Meeuwisse W, Dvorak J, et al Consensus statement on concussion in sport—the 5th international conference on concussion in sport held in Berlin October 2016. Br J Sports Med. 2017; 51:838–847. 28446457 10.1136/bjsports-2017-097699

[46] Covassin T , Elbin RJ, Stiller-Ostrowski JL, Kontos AP. Immediate post-concussion assessment and cognitive testing (ImPACT) practices of sports medicine professionals. J Athl Train. 2009;44:639–644. 19911091 10.4085/1062-6050-44.6.639 PMC2775366

[47] Sheehan DV , Sheehan KH, Shytle RD, et al Reliability and validity of the mini international neuropsychiatric interview for children and adolescents (MINI-KID). J Clin Psychiatry. 2010;71:313–326. 20331933 10.4088/JCP.09m05305whi

[48] Kaufman J , Birmaher B, Brent D, et al Schedule for affective disorders and schizophrenia for school-age children-present and lifetime version (K-SADS-PL): Initial reliability and validity data. J Am Acad Child Adolesc Psychiatry. 1997;36:980–988. 9204677 10.1097/00004583-199707000-00021

[49] Tottenham N , Tanaka JW, Leon AC, et al The NimStim set of facial expressions: Judgments from untrained research participants. Psychiatry Res. 2009;168:242–249. 19564050 10.1016/j.psychres.2008.05.006 PMC3474329

[50] Bates D , Mächler M, Bolker BM, Walker SC. Fitting linear mixed-effects models using lme4. J Stat Softw 2015;67:1–48.

[51] Kuznetsova A , Brockhoff PB, Christensen RHB. lmerTest package: Tests in linear mixed effects models. J Stat Softw. 2017;82:1–26.

[52] Makowski D . The psycho Package: An efficient and publishing-oriented workflow for psychological science. J Open Source Softw. 2018;3:470.

[53] Li X , Morgan PS, Ashburner J, Smith J, Rorden C. The first step for neuroimaging data analysis: DICOM to NIfTI conversion. J Neurosci Methods. 2016;264:47–56. 26945974 10.1016/j.jneumeth.2016.03.001

[54] Lutkenhoff ES , Rosenberg M, Chiang J, et al Optimized brain extraction for pathological brains (optiBET). PLoS One. 2014;9:e115551. 25514672 10.1371/journal.pone.0115551 PMC4267825

[55] Andersson JLR , Skare S, Ashburner J. How to correct susceptibility distortions in spin-echo echo-planar images: Application to diffusion tensor imaging. Neuroimage. 2003;20:870–888. 14568458 10.1016/S1053-8119(03)00336-7

[56] Jenkinson M , Bannister P, Brady M, Smith S. Improved optimization for the robust and accurate linear registration and motion correction of brain images. Neuroimage. 2002;17:825–841. 12377157 10.1016/s1053-8119(02)91132-8

[57] Jenkinson M , Smith S. A global optimisation method for robust affine registration of brain images. Med Image Anal 2001;5:143–156. 11516708 10.1016/s1361-8415(01)00036-6

[58] Andersson JLR , Jenkinson M, Smith S. Non-linear registration aka Spatial normalisation FMRIB Technial Report TR07JA2. 2007.

[59] Pruim RHR , Mennes M, van Rooij D, Llera A, Buitelaar JK, Beckmann CF. ICA-AROMA: A robust ICA-based strategy for removing motion artifacts from fMRI data. Neuroimage. 2015;112:267–277. 25770991 10.1016/j.neuroimage.2015.02.064

[60] Guillaume B , Hua X, Thompson PM, Waldorp L, Nichols TE. Fast and accurate modelling of longitudinal and repeated measures neuroimaging data. Neuroimage. 2014;94:287–302. 24650594 10.1016/j.neuroimage.2014.03.029 PMC4073654

[61] Vann SD , Aggleton JP, Maguire EA. What does the retrosplenial cortex do? Nat Rev Neurosci. 2009;10:792–802. 19812579 10.1038/nrn2733

[62] Miller AP , Vedder LC, Law ML, Smith DM. Cues, context, and long-term memory: The role of the retrosplenial cortex in spatial cognition. Front Hum Neurosci. 2014;8:586. 25140141 10.3389/fnhum.2014.00586 PMC4122222

[63] Gainotti G , Almonti S, Di Betta AM, Silveri MC. Retrograde amnesia in a patient with retrosplenial tumour. Neurocase. 1998;4:519–526.

[64] Todd TP , Mehlman ML, Keene CS, De Angeli NE, Bucci DJ. Retrosplenial cortex is required for the retrieval of remote memory for auditory cues. Learn Mem. 2016;23:278–288. 27194795 10.1101/lm.041822.116 PMC4880149

[65] Masuo O , Maeshima S, Kubo K, et al A case of amnestic syndrome caused by a subcortical haematoma in the right occipital lobe. Brain Inj. 1999;13:213–216. 10081602 10.1080/026990599121728

[66] Svoboda E , McKinnon MC, Levine B. The functional neuroanatomy of autobiographical memory: A meta-analysis. Neuropsychologia. 2006;44:2189–2208. 16806314 10.1016/j.neuropsychologia.2006.05.023 PMC1995661

[67] Maddock RJ . The retrosplenial cortex and emotion: New insights from functional neuroimaging of the human brain. Trends Neurosci. 1999;22:310–316. 10370255 10.1016/s0166-2236(98)01374-5

[68] Nestor PJ , Fryer TD, Ikeda M, Hodges JR. Retrosplenial cortex (BA 29/30) hypometabolism in mild cognitive impairment (prodromal Alzheimer’s disease). Eur J Neurosci. 2003;18:2663–2667. 14622168 10.1046/j.1460-9568.2003.02999.x

[69] Minoshima S , Giordani B, Berent S, Frey KA, Foster NL, Kuhl DE. Metabolic reduction in the posterior cingulate cortex in very early Alzheimer’s disease. Ann Neurol. 1997;42:85–94. 9225689 10.1002/ana.410420114

[70] Poirier GL , Amin E, Good MA, Aggleton JP. Early-onset dysfunction of retrosplenial cortex precedes overt amyloid plaque formation in Tg2576 mice. Neuroscience. 2011;3:71–83. 10.1016/j.neuroscience.2010.11.025 PMC4235255 21093545

[71] Keene CS , Bucci DJ. Damage to the retrosplenial cortex produces specific impairments in spatial working memory. Neurobiol Learn Mem. 2009;91:408–414. 19026755 10.1016/j.nlm.2008.10.009

[72] Manelis A , Huppert TJ, Rodgers E, Swartz HA, Phillips ML. The role of the right prefrontal cortex in recognition of facial emotional expressions in depressed individuals: fNIRS study. J Affect Disord. 2019;258:151–158. 31404763 10.1016/j.jad.2019.08.006 PMC6710146

[73] Bar M , Aminoff E. Cortical analysis of visual context from our findings, that parahippocampal and retrosplen-context-specific cortical processes. Neuron. 2003;38:347–358. 12718867 10.1016/s0896-6273(03)00167-3

[74] Maguire EA . The retrosplenial contribution to human navigation: A review of lesion and neuroimaging findings. Scand J Psychol. 2001;42:225–238. 11501737 10.1111/1467-9450.00233

[75] Mitchell AS , Czajkowski R, Zhang N, Jeffery K, Nelson AJD. Retrosplenial cortex and its role in spatial cognition. Brain Neurosci Adv. 2018;2:2398212818757098. 30221204 10.1177/2398212818757098 PMC6095108

[76] Fan F , Liao X, Lei T, et al Development of the default-mode network during childhood and adolescence: A longitudinal resting-state fMRI study. Neuroimage. 2021;226:117581. 33221440 10.1016/j.neuroimage.2020.117581

