# Supplementary material for: Vestibular/ocular motor symptoms in concussed adolescents are linked to retrosplenial activation
Source: Brain Commun. 2022 May 13;4(3):fcac123. doi: 10.1093/braincomms/fcac123 (PMC9127539; doi:10.1093/braincomms/fcac123)
Supplement: fcac123_Supplementary_Data [file fcac123_supplementary_data.docx]

# Supplementary material

Supplementary Table 1

Brain regions that increased in activation for 2-back vs. 1-back

| Cluster size | Local maxima Z-scores | x | y | z | Brain Regions |
| --- | --- | --- | --- | --- | --- |
| 22776 |  |  |  |  |  |
|  | 8.4 | 36 | -68 | -50 | Cerebellum |
|  | 8.12 | -30 | -66 | 38 | Lateral Occipital Cortex, superior division |
|  | 8.07 | -36 | -52 | 42 | Angular Gyrus |
|  | 8.02 | 32 | -58 | -32 | Cerebellum |
|  | 7.99 | -36 | -60 | 46 | Lateral Occipital Cortex, superior division |
|  | 7.92 | -28 | -68 | 50 | Lateral Occipital Cortex, superior division |
| 15562 |  |  |  |  |  |
|  | 7.62 | 0 | 22 | 48 | Paracingulate Gyrus |
|  | 7.57 | -30 | 2 | 54 | Middle Frontal Gyrus |
|  | 7.57 | -36 | 4 | 58 | Middle Frontal Gyrus |
|  | 7.51 | -4 | 24 | 48 | Superior Frontal Gyrus |
|  | 7.41 | -8 | 18 | 50 | Superior Frontal Gyrus |
|  | 7.3 | 24 | 4 | 54 | Superior Frontal Gyrus |
| 2905 |  |  |  |  |  |
|  | 8 | -30 | 22 | -2 | Insular Cortex |
|  | 6.91 | -14 | 2 | 18 | Left Caudate |
|  | 6.14 | 10 | 8 | 12 | Right Caudate |
|  | 6 | 12 | 4 | 18 | Right Caudate |
|  | 5.56 | -10 | -10 | 10 | Left Thalamus |
|  | 5.31 | 10 | -4 | 6 | Right Thalamus |
| 491 |  |  |  |  |  |
|  | 7.07 | 32 | 22 | 0 | Insular Cortex |
|  | 7.05 | 36 | 22 | -2 | Insular Cortex |
|  | 6.79 | 38 | 22 | -6 | Frontal Orbital Cortex |
|  | 6.13 | 30 | 26 | -6 | Frontal Orbital Cortex |
| 338 |  |  |  |  |  |
|  | 5.05 | 4 | -16 | -18 | Brain-Stem |
|  | 4.93 | -8 | -30 | -14 | Brain-Stem |
|  | 3.88 | 0 | -32 | -18 | Brain-Stem |
|  | 3.62 | -10 | -18 | -16 | Brain-Stem |
| 317 |  |  |  |  |  |
|  | 4.82 | 64 | -34 | -14 | 52% Middle Temporal Gyrus, posterior division |
|  | 4.19 | 62 | -36 | -8 | 29% Middle Temporal Gyrus, posterior division |
|  | 3.62 | 70 | -42 | -8 | Middle Temporal Gyrus, temporooccipital part |
|  | 3.33 | 64 | -18 | -16 | Middle Temporal Gyrus, posterior division |
| 132 |  |  |  |  |  |
|  | 4.04 | -54 | -56 | -12 | Inferior Temporal Gyrus, temporooccipital part |
|  | 3.28 | -50 | -50 | -4 | Middle Temporal Gyrus, temporooccipital part |

Supplementary Table 2

Brain regions that decreased in activation for 2-back vs. 1-back

| Cluster size | Local maxima Z-scores | x | y | z | Brain Regions |
| --- | --- | --- | --- | --- | --- |
| 59387 |  |  |  |  |  |
|  | 7.93 | 4 | -6 | 40 | Cingulate Gyrus, anterior division |
|  | 7.59 | -2 | -2 | 40 | Cingulate Gyrus, anterior division |
|  | 7.5 | 56 | 2 | 4 | Central Opercular Cortex |
|  | 7.48 | -6 | 2 | 42 | Cingulate Gyrus, anterior division/ Juxtapositional Lobule Cortex |
|  | 7.35 | -48 | -28 | 18 | Parietal Operculum Cortex |
|  | 7.23 | -8 | -22 | 42 | Cingulate Gyrus, posterior division |
